# Free Space Estimation Based on Superpixel Clustering for Assisted Driving

**DOI:** 10.3390/s26072120

**Published:** 2026-03-29

**Authors:** Oswaldo Vitales, Ruth Aguilar-Ponce, Javier Vigueras

**Affiliations:** Facultad de Ciencias, Universidad Autónoma de San Luis Potosí, 1570 Parque Chapultepec Ave, San Luis Potosí 78295, Mexico; rmariela@fciencias.uaslp.mx (R.A.-P.); javier.vigueras@uaslp.mx (J.V.)

**Keywords:** driving free space, assisted driving, superpixel, coplanarity, SLIC, census transform, semi-global matching

## Abstract

Free space detection in assisted driving applications is essential to provide information to vehicles about traversable surfaces and potential obstacles to be avoided. The current trend in free space detection favors the use of deep learning techniques. However, Deep Neural Networks require extensive training that considers as many scenarios as possible, which makes it difficult to create a model that can be generalized to all types of surfaces. Additionally, their lack of explainability contrasts with the growing interest in geometrically grounded and safety-oriented design principles for autonomous vehicle systems. To address these limitations, we propose a geometric approach that incorporates coplanarity conditions and normal vector estimation, removing the dependence on datasets for different types of surfaces. Additionally, the stereoscopic images are clustered in superpixels. The use of images clustered in superpixels allows us to obtain shorter processing times, in addition to taking advantage of the spatial and color information provided by the superpixels to increase the robustness of the three-dimensional reconstruction of the scene. Experimental results show that the proposed superpixel-based approach achieves competitive performance compared to unsegmented dense stereo methods, while significantly reducing algorithmic complexity. These results demonstrate the viability of integrating superpixel clustering into stereo-based free space estimation frameworks.

## 1. Introduction

In recent years, autonomous vehicles have made fast progress thanks to advances in areas such as stereoscopy, artificial intelligence and computer vision. To achieve safe and efficient driving, these vehicles must perceive their surroundings (identifying ground, vehicles, and pedestrians) to plan obstacle-free trajectories. In this context, algorithms for estimating free space, defined as the navigable area through which a vehicle can safely travel [1], are essential for integrating surface identification and risk detection into a single process.

The primary technologies used for environmental perception in autonomous systems are stereoscopic vision and Light Detection and Ranging (LiDAR) sensors. Both modalities present complementary advantages and limitations. LiDAR systems provide accurate depth measurements and are less sensitive to illumination changes; however, they may involve higher costs and specific hardware constraints. On the other hand, stereoscopic vision offers a cost-effective alternative capable of delivering dense three-dimensional (3D) information directly from image data, although its performance can degrade under adverse weather conditions, low illumination, or textureless regions [2,3]. In addition, previous studies have also reported potential vulnerabilities in LiDAR-based perception systems by injecting false data through deceptive laser point clouds [4]. This paper therefore focuses on stereoscopic cameras as a cost-effective solution for dense 3D perception in autonomous driving scenarios.

In stereo-based free space estimation, the major challenges include disparity noise in low-texture regions, depth discontinuities near object boundaries, sensitivity to lighting changes, and the high computational cost of dense matching algorithms such as Semi-Global Matching (SGM). Furthermore, many geometric methods assume flat terrain or strict gravitational alignment, which limits their robustness in scenarios with slopes or irregular surfaces.

To address these challenges, Deep Neural Networks (DNNs) have been widely employed in assisted driving applications and have achieved competitive performance. However, a key limitation of these approaches is their reliance on large, labeled datasets for training. Acquiring such datasets is a resource-intensive process that must capture the dynamic variability of real-world driving environments, including changes in illumination, weather conditions, and occlusions. As a result, data scarcity and high annotation costs remain significant challenges. Furthermore, DNN-based systems often exhibit limited generalization across diverse terrains (e.g., urban potholes versus rural gravel roads) due to domain shifts, whereby models trained on a particular dataset tend to underperform when deployed in previously unseen conditions unless they are continually retrained. These limitations have motivated the development of alternative systems grounded in explicit geometric principles, in which vehicle decisions are supported by well-defined mathematical models that promote more interpretable and controllable behavior. In addition, DNN-based methods remain vulnerable to adversarial attacks, where subtle perturbations in input images can lead to incorrect traversability predictions, potentially compromising safety in adversarial scenarios such as manipulated or hacked traffic signs [5].

In response to these limitations, a free space estimation system based on a geometric approach is proposed, in which the selection criterion is based on the coplanarity condition of the elements belonging to the ground. Additionally, image grouping into superpixels is used to perform 3D reconstruction and adjust the ground plane to the road, with the aim of reducing processing time per frame and improving the spatial consistency of the disparity.

The main contribution of this work lies in a free space estimation framework that integrates superpixel-based disparity reduction with robust coplanarity constraints. Unlike previous approaches that either rely on dense pixel-wise disparity or computationally intensive free space labeling optimization, the proposed method incorporates superpixel clustering directly into the disparity computation, reducing computational complexity while maintaining geometric consistency.

To evaluate the effectiveness of using images grouped into superpixels, comparisons are conducted against a methodology based on unsegmented images using Semi-Global Matching (SGM) [6] with Census Transform (CT) [7] for 3D reconstruction and subsequent free space detection via coplanarity. Additionally, SegFormer-B0 [8], which addresses free space detection through semantic segmentation, is included as a reference method.

The rest of this paper is organized as follows: Section 2 summarizes the recent methodologies most commonly used in the literature for free space estimation. Section 3 shows the details of the methodology proposed in this work for free space detection. Section 4 shows the results obtained by implementing the methodology and shows a comparison with SGM and SegFormer-B0. Finally, Section 5 presents the discussion of the results and proposes topics for future work.

## 2. Related Work

In recent years, several methods have been proposed to estimate free space using stereoscopic vision systems. Most stereo-based approaches rely on Semi-Global Matching (SGM) combined with the Census Transform (CT) for disparity computation. Therefore, SGM with CT is adopted in this work as the reference baseline, as it remains one of the most widely used stereo matching methods and serves as a representative benchmark for many geometric free-space estimation techniques (including plane-labeling and stixel-based approaches).

Based on these foundations, free space detection methods can be broadly categorized into learning-based, geometric, and sensor fusion approaches.

Convolutional Neural Networks (CNNs) are currently among the most widely used methods for ground detection, typically formulated as binary or multi-class semantic segmentation that distinguishes between traversable and non-traversable terrain. These techniques have demonstrated competitive accuracy and processing speeds of up to 19 frames per second [9,10,11]. However, their main limitation lies in their strong dependence on large and diverse training datasets that capture variations in weather conditions, terrain inclinations, illumination changes, and obstacle types. Constructing such datasets is both challenging and costly, which may limit their deployment flexibility in unseen environments.

To mitigate these limitations, sensor fusion strategies are widely employed in autonomous driving systems. Several frameworks combine stereo vision with LiDAR, radar, or inertial sensors to improve robustness and perception accuracy under challenging conditions [12,13]. Fusion-based methods leverage complementary sensing modalities to compensate for weaknesses in individual sensors, particularly in low-texture regions or adverse weather scenarios. While these approaches enhance reliability, they increase system complexity, hardware cost, energy consumption, and calibration and synchronization requirements, as well as end-to-end latency (from sensors to actuators) [14]. In contrast, stereo-only systems remain attractive for low-cost and compact implementations, motivating research on improving geometric robustness within monocular or stereo setups.

Among geometric approaches, Maximally Stable Extremal Regions (MSER) have been applied for ground detection by identifying stable intensity regions in the image [15]. This method selects a seed point near the bottom edge of the image under the assumption that the road begins there. Borders are then extracted to determine closed regions corresponding to the ground. Although computationally simple, this approach strongly depends on the correctness of the initial seed assumption, which may not hold in complex or unstructured environments.

Stixel-based representations constitute another geometric alternative. Stixels represent the environment as vertical rectangular elements derived from a disparity image. A V-disparity map is constructed, and a line-fitting algorithm estimates the ground plane. Obstacles are detected as pixels whose disparity significantly deviates from the estimated ground [16]. This method provides a compact representation and reduces data dimensionality, enabling efficient obstacle detection. However, it reduces terrain detail resolution, assumes predominantly vertical obstacle structures, and relies heavily on accurate initial ground plane estimation. Furthermore, irregular or sloped surfaces are difficult to model, and terrain stixels may occlude nearby objects.

Superpixel-based approaches have also been explored to improve spatial coherence and reduce computational complexity. In [17], a disparity map is first computed using SGM, followed by normal vector estimation for each superpixel. These normals are clustered to identify ground regions. Although effective in reducing errors in low-texture areas, computing a normal vector per superpixel introduces additional computational overhead.

A closely related approach was presented in [18], where superpixels were used to refine previously computed disparity maps through a global optimization framework based on Markov Random Fields (MRF). This method improves depth propagation in incomplete or noisy regions but introduces a computationally expensive post-processing stage due to energy minimization and iterative refinement.

Similarly, Ref. [19] employs superpixels as planar elements in a continuous plane-labeling formulation. Each superpixel is associated with a 3D plane and optimized through a hierarchical coarse-to-fine strategy enforcing geometric consistency. While this results in highly accurate disparity estimation, it requires complex optimization procedures, multiple parameters, and significant computational resources.

In contrast to these approaches, the methodology proposed in this work directly integrates superpixels into the disparity computation stage. Superpixels are used as irregular support regions within the Census Transform, assigning a representative disparity value per region and incorporating color similarity in the CIELAB space, followed by a lightweight local refinement step. This design reduces algorithmic complexity and avoids global optimization frameworks, while preserving geometric consistency and robustness in low-texture regions.

From the reviewed literature, several common limitations can be identified:

- Strong geometric assumptions, such as vertical obstacle models or fixed road initialization, restrict applicability in irregular or sloped terrains.
- Dependence on large and diverse datasets limits the generalization capacity of learning-based approaches.
- Normal vector estimation per region and global optimization frameworks introduce significant computational overhead.
- Multi-stage pipelines separating disparity estimation, global refinement, and post-processing increase implementation complexity and parameter tuning requirements.
- Hierarchical optimization strategies may propagate errors across scales in ambiguous or low-texture regions.
- Sensor fusion systems, while robust, increase hardware cost and calibration complexity.

The proposed approach addresses these limitations by adopting a geometrically grounded framework that reduces computational complexity through superpixel-based disparity estimation while avoiding restrictive terrain assumptions and expensive global optimization stages. Rather than relying on dense pixel-wise disparity or multi-stage refinement, the method integrates region-based disparity estimation with coplanarity constraints derived from stereo geometry. The following section describes the mathematical formulation and implementation details of this framework.

## 3. Methodology

The proposed methodology is based on the geometric relationship between stereo disparity and planar surfaces in three-dimensional space. In particular, free space is identified by exploiting the coplanarity condition of points belonging to the ground surface. To reduce computational complexity while preserving spatial coherence, the reference image is first segmented into superpixels, which serve as irregular support regions for disparity estimation.

Instead of computing dense pixel-wise disparity across the entire image, a representative disparity value is assigned to each superpixel based on Census Transform matching. This regional formulation decreases the number of matching operations and improves robustness in low-texture areas. Once the disparity map is obtained, a ground plane model is estimated using a robust optimization scheme, allowing for adaptation to sloped and non-uniform terrains without imposing strict flat-ground assumptions.

The overall pipeline consists of three main stages: (1) superpixel segmentation, (2) superpixel-based disparity estimation, and (3) ground plane fitting and free space extraction. Each stage is described in detail in the following subsections.

### 3.1. Homography Induced by a Plane in a Stereo Rig

**Figure 1:**
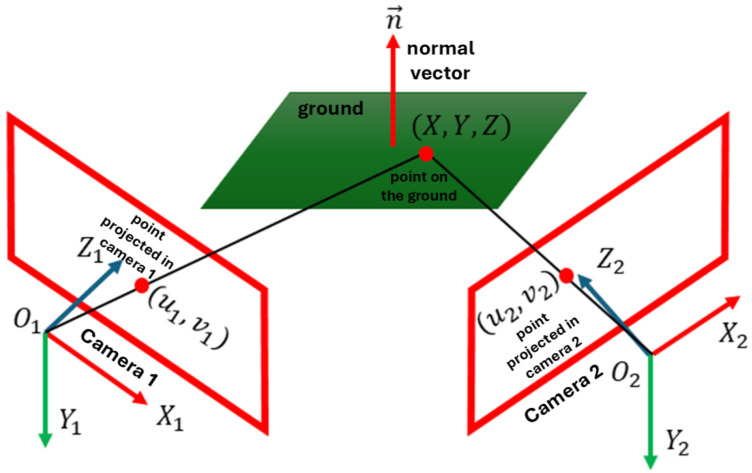
Relationship between the coordinate of the point (X,Y,Z) belonging to a plane with normal vector $\vec{n}$ and the coordinates $\left(u_{1},v_{1}\right)$ and $\left(u_{2},v_{2}\right)$ of the projected point in both images with $O_{1}$ and $O_{2}$ as optical centers of each camera respectively.

If we consider only the relationship between image point coordinates $\left(u_{1},v_{1}\right)$ and $\left(u_{2},v_{2}\right)$ of each point of a plane observed in two images (as shown in Figure 1), we obtain a homography induced by a plane. According to [20], the expression defining homography induced by a plane is shown in (1).

(1) $$\left(\begin{array}{c} u_{2}\\ v_{2}\\ 1 \end{array}\right)∼K_{2}\left(R-\frac{1}{d}\vec{t}\cdot {\vec{n}}^{T}\right)K_{1}^{-1}\left(\begin{array}{c} u_{1}\\ v_{1}\\ 1 \end{array}\right)=H_{p}\left(\begin{array}{c} u_{1}\\ v_{1}\\ 1 \end{array}\right)$$

where $\vec{n}$ is the normal vector to the plane being captured, $K_{1}$ and $K_{2}$ are the intrinsic parameter matrices of each camera, respectively, *R* is the rotation matrix, $\vec{t}$ is the translation vector, *d* is the orthogonal distance of the plane from the first camera, $H_{p}$ is the homography induced by a plane, and the expression $R-\frac{1}{d}\vec{t}{\vec{n}}^{T}$ represents the collineation matrix.

It should be noted that $H_{p}$ corresponds to a particular case of the general homography matrix. (1) expresses the homography induced specifically by the ground plane, parameterized in terms of the stereo configuration and plane parameters, describing a general homography between two views.

The mathematical model of calibrated and rectified stereoscopic cameras implies that the rotation is the identity matrix and that the translation is just a displacement through the x-axis. Additionally, as the cameras are calibrated, their intrinsic parameter matrices are supposed to be known (see (2a) and (2b)).

(2a) $$R=\left(\begin{array}{ccc} 1 & 0 & 0\\ 0 & 1 & 0\\ 0 & 0 & 1 \end{array}\right);\;\;\;\;\;\vec{t}=\left(\begin{array}{c} t_{x}\\ 0\\ 0 \end{array}\right)$$

(2b) $$K_{1}=\left(\begin{array}{ccc} f_{1} & 0 & u_{01}\\ 0 & f_{1} & v_{01}\\ 0 & 0 & 1 \end{array}\right);\;\;\;\;\;K_{2}=\left(\begin{array}{ccc} f_{2} & 0 & u_{02}\\ 0 & f_{2} & v_{02}\\ 0 & 0 & 1 \end{array}\right)$$

where $f_{1}$ and $f_{2}$ are the focal lengths of each camera, $\left(u_{01},v_{01}\right)$ and $\left(u_{02},v_{02}\right)$ are the coordinates of the principal point (where the focal axis intersects with the image plane) of each camera, and $t_{x}$ is the distance between the two camera centers.

If we consider the ground as a horizontal plane, the gravity condition is satisfied, i.e., with a horizontal plane, its normal vector must be completely vertical with respect to the camera coordinate system, so that $\vec{n}={\left(\begin{array}{ccc} 0 & 1 & 0 \end{array}\right)}^{T}$, where the expression of the homography induced by a plane would be as shown in (3). It is important to note that this assumption is only employed as an initialization step to obtain an initial estimate of the ground surface closest to the vehicle. The estimated plane parameters are subsequently refined through the coplanarity constraints, and in successive frames the normal vector obtained from the previous estimation is used as the reference. Therefore, the method does not impose a permanent horizontal constraint and can adapt to inclined or non-planar terrains.

(3) $$H_{p}=\left(\begin{array}{ccc} \frac{f_{2}}{f_{1}} & -\frac{f_{2}t_{x}}{f_{1}d} & \left(\frac{f_{2}}{f_{1}}\right)\left(\frac{t_{x}}{d}v_{01}-u_{01}\right)+u_{02}\\ 0 & \frac{f_{2}}{f_{1}} & -\left(\frac{f_{2}}{f_{1}}\right)v_{01}+v_{02}\\ 0 & 0 & 1 \end{array}\right)$$

### 3.2. Coplanarity Condition

We consider using the coplanarity condition to eliminate the restriction of the gravity condition to planes that are only parallel to the XZ plane of the camera coordinate system. This allows us to detect surfaces with considerable inclinations, unlike methods where the ground is considered to be flat [21].

Several points are considered coplanar if and only if they all belong to the same plane in three-dimensional space. When we calculate the homography induced by a plane to two images, we are relating corresponding points in both images that come from the same plane in space. Then, by studying the homography between images, we can infer the three-dimensional structure of a scene, including the coplanarity of points.

Thus, knowing that the homography induced by a plane $H_{p}$ corresponds to the relationship that both cameras have across the ground plane, then the relationship is expressed as shown in (4).

(4) $$\left(\begin{array}{c} u_{2}\\ v_{2}\\ 1 \end{array}\right)∼\left(\begin{array}{ccc} \frac{f_{2}}{f_{1}} & -\frac{f_{2}t_{x}}{f_{1}d} & \left(\frac{f_{2}}{f_{1}}\right)\left(\frac{t_{x}}{d}v_{01}-u_{01}\right)+u_{02}\\ 0 & \frac{f_{2}}{f_{1}} & -\left(\frac{f_{2}}{f_{1}}\right)v_{01}+v_{02}\\ 0 & 0 & 1 \end{array}\right)\left(\begin{array}{c} u_{1}\\ v_{1}\\ 1 \end{array}\right)$$

Solving for $u_{2}-u_{1}$ which corresponds to the disparity, i.e., the difference in pixels between both images of each coordinate with respect to its horizontal axis, we obtain (5a).

(5a) $$u_{2}-u_{1}=\left(\frac{f_{2}}{f_{1}}\right)u_{1}-\left(\frac{f_{2}t_{x}}{f_{1}d}\right)v_{1}+\left(\frac{f_{2}}{f_{1}}\right)\left(\left(\frac{t_{x}}{d}\right)v_{01}-u_{01}\right)+u_{02}$$

here:

(5b) $$\begin{array}{c} x=u_{1};\;\;\;\;\;y=v_{1};\;\;\;\;\;z=u_{2}-u_{1};\;\;\;\;\;a=\left(\frac{f_{2}}{f_{1}}\right); \end{array}$$

(5c) $$\begin{array}{c} b=-\left(\frac{f_{2}t_{x}}{f_{1}d}\right);\;\;\;\;\;c=\left(\frac{f_{2}}{f_{1}}\right)\left(\left(\frac{t_{x}}{d}\right)v_{01}-u_{01}\right)+u_{02} \end{array}$$

where for all ternary $\left(u_{i},v_{i},d_{i}\right)$ we have $ax_{i}+by_{i}-z_{i}+c=0$. Then, for *i* correspondences we use the pixel coordinates and their respective disparity to compute an estimate of a plane passing through all these values, minimizing the mean squared error according to (6).

(6) $$\left(\begin{array}{ccc} x_{1} & y_{1} & 1\\ x_{2} & y_{2} & 1\\ \vdots & \vdots & \vdots \end{array}\right)\left(\begin{array}{c} a\\ b\\ c \end{array}\right)=\left(\begin{array}{c} z_{1}\\ z_{2}\\ \vdots \end{array}\right)$$

where for *i* pixels, $z_{i}$ corresponds to the disparity $d_{i}$ of each pixel, $\left(x_{i},y_{i}\right)$ are the pixel coordinates $\left(u_{i},v_{i}\right)$, and the vector ${\left(a,b,c\right)}^{T}$ is the normal vector to the calculated plane.

Then, according to (6), we can obtain a plane that fits the ground from a disparity map. In this work, we propose using disparity maps based on images clustered into superpixels to reduce computational cost, using the coordinates of each centroid as $\left(x_{i},y_{i}\right)$ and its corresponding disparity value as $z_{i}$.

For this reason, before obtaining the disparity map, it is necessary to perform the segmentation of one of the stereoscopic images.

### 3.3. Image Segmentation

It is important to emphasize that the segmentation step corresponds to superpixel clustering rather than semantic segmentation. The goal is to obtain compact and spatially coherent regions that facilitate disparity estimation and reduce computational complexity, without requiring object-level classification.

The left image was segmented into superpixels. Segmentation groups pixels with similar color and spatial characteristics, which typically correspond to locally coherent regions. Although pixels within a superpixel are not guaranteed to share identical depth values, the controlled size of the superpixels limits intra-region disparity variation. Therefore, a representative disparity value can be assigned to each superpixel with minimal approximation error.

It is important to note that this approximation may introduce boundary errors in regions with sharp depth discontinuities. However, for the purpose of free space detection, small disparity variations within a superpixel do not significantly alter the classification between ground and obstacles. The coplanarity constraint evaluates geometric consistency at the plane level, which mitigates the impact of minor local disparity differences.

The Simple Linear Iterative Clustering (SLIC) algorithm is adopted because it produces compact and regular superpixels with low computational cost, while maintaining segmentation quality comparable to more complex methods [22,23]. In this work, superpixel segmentation is used primarily to reduce the computational complexity of disparity estimation by grouping pixels with similar characteristics into homogeneous regions. For this reason, we intentionally rely on a lightweight segmentation strategy. Employing more complex approaches, such as semantic segmentation, would introduce additional computational overhead that is not required for the objective of this stage and could offset the efficiency gains provided by the superpixel-based representation.

SLIC operates in a five-dimensional space [labxy], combining CIELAB color values and spatial coordinates. This defines a distance metric $D_{s}$ that incorporates color similarity and spatial proximity:

(7a) $$d_{lab}=\sqrt{{\left(l_{k}-l_{i}\right)}^{2}+{\left(a_{k}-a_{i}\right)}^{2}+{\left(b_{k}-b_{i}\right)}^{2}}$$

(7b) $$d_{xy}=\sqrt{{\left(x_{k}-x_{i}\right)}^{2}+{\left(y_{k}-y_{i}\right)}^{2}}$$

(7c) $$S=\sqrt{\frac{N}{K}}$$

(7d) $$D_{s}=d_{lab}+\frac{m}{S}d_{xy}$$

where *N* is the number of pixels, *K* is the number of desired superpixels, *S* is the grid interval between cluster centers, *m* controls compactness, and $C_{k}={\left[l_{k},a_{k},b_{k},x_{k},y_{k}\right]}^{T}$ is the center of a cluster. Subsequently, using the segmented image, we can obtain the disparity map.

### 3.4. Disparity Calculation Based on Superpixel Clustering

In our previous work [24], we proposed a methodology that computes disparity using superpixels, which we adopt in this study. The image is segmented using SLIC, and then a modified Census Transform (CT) is applied. Due to the irregular superpixel shapes, the original CT is adapted for non-rectangular regions.

**Figure 2:**
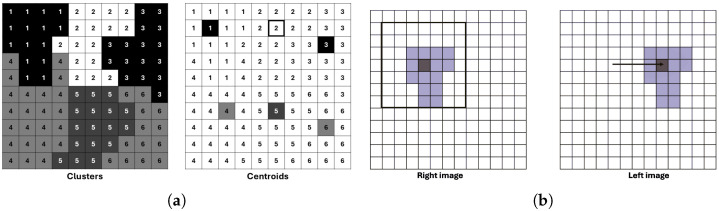
Examples illustrating the SLIC segmentation process and its application in stereo matching. (**a**) Image segmented into pixel clusters and the corresponding cluster centroids. (**b**) Determination of the search window size for a pixel cluster in the right stereo image and its correspondence search in the left image for different disparity values.

We assume that disparity is constant within each superpixel, so we compute disparity only at the superpixel centroid (Figure 2a), significantly reducing computation. A search window of size 4S×4S is defined around each centroid to determine the best disparity match (Figure 2b).

In addition to the Hamming distance from CT, we incorporate a perceptually accurate color difference in CIELAB space. The combined cost function is

(8a) $$C=w_{c}\cdot {\hat{d}}_{H}+\left(1-w_{c}\right)\cdot {\hat{d}}_{lab}$$

with normalized terms

(8b) $${\hat{d}}_{H}=\frac{\sum CT_{k}⨂CT_{k}^{\prime }}{n}$$

(8c) $${\hat{d}}_{lab}=\frac{\sum_{\forall i}\sqrt{{\left(l_{k}-l_{i}\right)}^{2}+{\left(a_{k}-a_{i}\right)}^{2}+{\left(b_{k}-b_{i}\right)}^{2}}}{12n}$$

where CT and $CT^{\prime }$ are binary descriptors from the left and right images, *n* is the number of pixels in a superpixel, and $w_{c}\in \left[0,1\right]$ weights the two components. $\left[l_{k},a_{k},b_{k}\right]$ is the vector lab of the cluster center and $\left[l_{i},a_{i},b_{i}\right]$ is the vector lab of each pixel belonging to the cluster. The normalization factor 12 corresponds to a perceptually significant color difference [25].

### 3.5. Determination of Superpixels That Belong to the Ground Plane (Influences)

The disparity map is used to estimate the ground plane. However, as not all pixels belong to the ground, outliers must be excluded to improve estimation accuracy. As a heuristic, we use the assumption that vanishing points lie near the image center after calibration [26], and restrict candidate pixels to those below the image center. A maximum likelihood-type estimator, or M-estimator, can be used for its robustness to outliers. Robust estimators utilize influence functions to reduce the impact of outliers on the estimation process. A commonly used robust estimator is the Cauchy loss function:

(9) $$\rho \left(r\right)=\frac{c^{2}}{2}\ln\left(1+{\left(\frac{r}{c}\right)}^{2}\right)$$

Here, *r* is the residual and *c* is a tuning constant. The Cauchy function reduces the influence of large residuals without completely discarding them, making it effective in scenarios with moderate to strong outliers.

The Iteratively Reweighted Least Squares (IRLS) method is applied to this M-estimator [27]. The IRLS updates the solution at each iteration using the weighted least squares approach:

(10) $$\min_{\vec{\theta }}\sum_{i=1}^{n}w\left(r_{i}\right)\cdot {\left(y_{i}-f\left(x_{i}\right)\right)}^{2}$$

The weights $w(r_{i})$ are derived from the derivative of the influence function:

(11) $$w\left(r_{i}\right)=\frac{1}{1+{\left(\frac{r_{i}}{c}\right)}^{2}}$$

These weights are updated iteratively until convergence. This yields a more robust estimation of the ground plane, especially in the presence of obstacles or noise.

### 3.6. Free Space Estimation

Once the ground plane is estimated, we identify superpixels that lie on or above it. Superpixels are classified based on the vertical distance between their centroid and the estimated plane. Those whose disparity-derived depth lies within a small threshold of the plane are considered part of the free space. It is important to mention that although, initially, the gravity condition is considered as shown in (3), the normal vector to the surface is updated to adapt to different inclinations, as shown in (6), so the estimated plane would be that shown in (12).

(12) z=ax+by+c

A superpixel with centroid $\left(x_{s},y_{s},z_{s}\right)$ is considered part of the ground if

(13) $$|z_{s}-\left(ax_{s}+by_{s}+c\right)|<\epsilon$$

Here, ϵ is a small threshold that accounts for noise and estimation uncertainty.

To further refine the free space detection, we enforce spatial consistency by checking whether neighboring superpixels also satisfy the above condition. This helps reduce isolated misclassifications caused by noise or occlusions.

Finally, the regions corresponding to ground superpixels are merged to define the drivable area or free space. This output can be overlaid on the image to provide a visual representation of safe navigation zones.

### 3.7. Algorithm

The implementation of the proposed system is formalized in Algorithm 1. The process begins with the segmentation of the reference image into superpixels using the SLIC method, followed by an initial Winner-Takes-All (WTA) disparity assignment. To improve robustness against lack of texture, the matching cost combines intensity differences in the CIELAB color space with Laplacian gradient information. A critical component of our pipeline is the post-processing stage, where we apply an Iteratively Reweighted Least Squares (IRLS) optimization to fit a geometric model to the ground plane. This allows for the generation of a specific soil mask, which is subsequently used to regularize the disparity values in the lower regions of the image, effectively mitigating noise caused by perspective distortion and lack of texture.

**Algorithm 1:** Disparity Stereo Matching
**Input:** Left and Right Images $\left\{I_{L},I_{R}\right\}$, Superpixel count *K*, Thresholds σ,τ.
**Output:** Disparity Map *D*, Ground Soil Mask $M_{soil}$.

**1: Initialization and Pre-processing**

   1.1: Apply Gaussian Blur (5×5) to $I_{L}$ and $I_{R}$.
   1.2: Generate superpixels $S=\{S_{1},\ldots ,S_{K}\}$ on $I_{L}$ using SLIC.
   1.3: Compute CIELAB color space.

**2: Initial Disparity Estimation**

   **for** each superpixel $S_{k}\in S$ **do**
      Define search range $d\in [0,d_{max}]$.
      Compute cost C(d) combining Color and Gradient differences:
         $C\left(d\right)=w_{c}\cdot C_{grad}+\left(1-w_{c}\right)\cdot C_{color}$.
      $D\left(S_{k}\right)\leftarrow \arg\min_{d}C\left(d\right)$.
   **end for**

**3: Spatial Filtering (Propagation)**

   **repeat** 5 times
      Update $D(S_{k})$ by minimizing energy with 8-connected neighbors.
      Handle occlusions by checking consistency.
   **end repeat**

**4: Robust Ground Plane Estimation (IRLS)**

   4.1: Select seed superpixels $P_{seed}$ from the lower image region.
   4.2: Initialize plane parameters $w={\left[w_{0},w_{1},w_{2}\right]}^{T}$.
   **for** t=1 to 50 **do**
      Construct matrix A (coordinates) and B (disparities) from seeds.
      Compute residuals $r_{i}$ between observed disparity and plane model.
      Update weights $\omega_{i}$ using robust function (e.g., Cauchy):
         $\omega_{i}\leftarrow {\left(1+{\left(r_{i}/\sigma \right)}^{2}\right)}^{-2}$.
      Solve Weighted Least Squares: $w\leftarrow {\left(A^{T}WA\right)}^{-1}A^{T}WB$.
   **end for**

**5: Mask Refinement and Projective Correction**

   5.1: Generate binary mask $M_{soil}$ where |D(x)−Plane(x,w)|<τ.
   5.2: Apply morphological opening and closing to refine $M_{soil}$.
   5.3: **for** each pixel *x* where $M_{soil}\left(x\right)==1$ **do**
      Overwrite D(x) with projected plane value: $D\left(x\right)\leftarrow w\cdot {\left[u,v,1\right]}^{T}$.
      **end for**
**6: Return** $D,M_{soil}$


## 4. Results

This section presents the results obtained after applying the methodology described in the previous section. It is important to mention that the KITTI Stereo Evaluation 2015 benchmark [28], composed of 200 scenes (stored in lossless PNG format with a resolution of 1242×375), has been used to perform all the calculations. This dataset comprises dynamic scenes for which the ground truth has been established through a semi-automatic process.

In order to extend the experimental validation beyond the KITTI dataset and evaluate robustness under diverse environmental conditions, an additional evaluation was conducted using the DrivingStereo dataset [29]. For this purpose, a subset designed for image restoration tasks was selected, consisting of 2000 frames with different weather conditions (saved in jpg format with resolution 881×400). Specifically, the dataset includes 500 frames per weather category: sunny, cloudy, foggy, and rainy. This dataset introduces significant challenges, such as illumination changes, visibility degradation, and atmospheric artifacts, allowing for a more comprehensive analysis of the behavior of the evaluated methods under real-world conditions.

The tests were performed on a computer with a Ryzen 3 processor, 8 GB of RAM, and the Windows 11 operating system. The algorithms were implemented in C++ using the OpenCV 4.0 library [30], an open-source library designed for image processing and computer vision.

### 4.1. Image Segmentation Results

**Figure 3:**
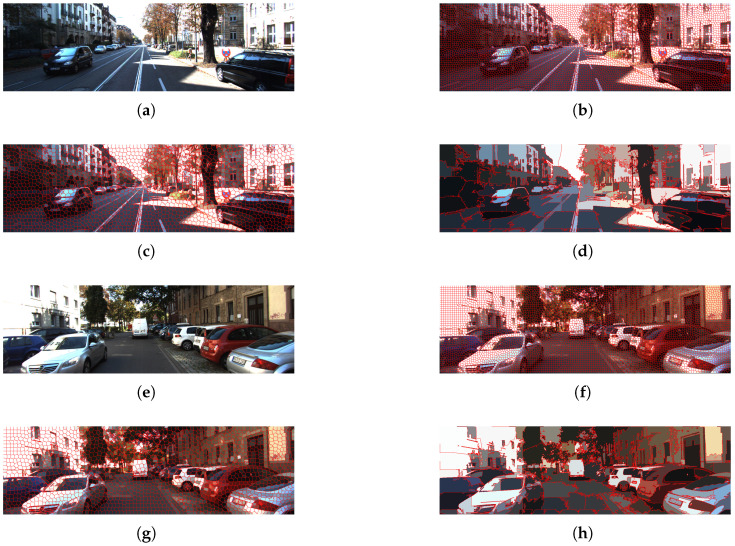
Comparison of segmentation results for different SLIC window sizes. (**a**,**e**) Original KITTI images; (**b**,**f**) window size 9×9; (**c**,**g**) 15×15; (**d**,**h**) 75×75.

In this case, the left image was selected for segmentation, and the SLIC algorithm is used, as described in Section 3. Two frames with different initial sizes of SLIC windows are shown in Figure 3: 9×9, 15×15 and 75×75 respectively. Figure 3 shows how as the SLIC window sizes increase the pixels are grouped into larger segments.

**Figure 4:**
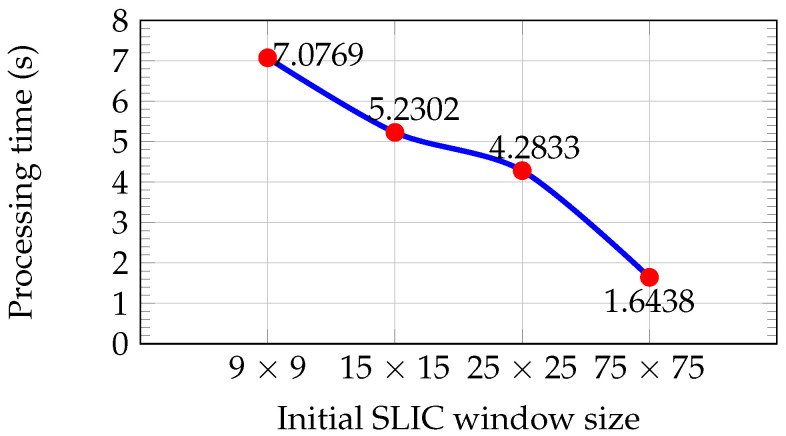
Average processing time for segmentation versus initial SLIC window size for the set of 200 KITTI images.

Figure 4 shows the average processing time for each initial window size, computed from the set of 200 KITTI images until a residual error convergence of 1% is reached. Larger window sizes reduce execution time due to the lower number of superpixels and iterations required.

If only processing time is considered, larger window sizes are preferable due to their lower computational cost (see Figure 4). However, as illustrated in Figure 3d,h, larger superpixels lead to a loss of important details such as road boundaries, increasing the risk of missing small obstacles. In contrast, window sizes of 9×9 and 15×15 preserve these details (see Figure 3b,c,f,g). The window size of 15×15 provides the best trade-off between information preservation and processing time and was therefore selected for segmenting the left image of the stereo pair. This choice is also consistent with typical support window sizes used in the Census Transform descriptor.

### 4.2. Disparity Map Calculation

In our previous work [24], the values of $w_{c}$, $P_{1}$, and $P_{2}$ that minimize the average percentage error were determined using the KITTI dataset by comparing the estimated disparity maps with the ground truth. A pixel was considered correctly estimated if the disparity error was less than 5 pixels.

**Figure 5:**
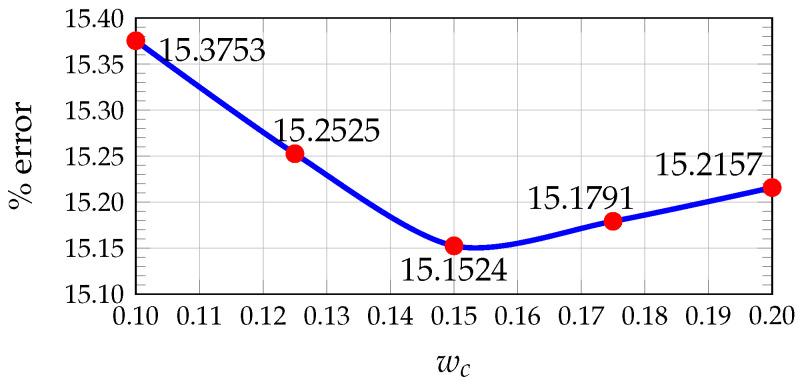
Plot of average percentage error vs. Hamming weight value for 200 images from KITTI.

**Figure 6:**
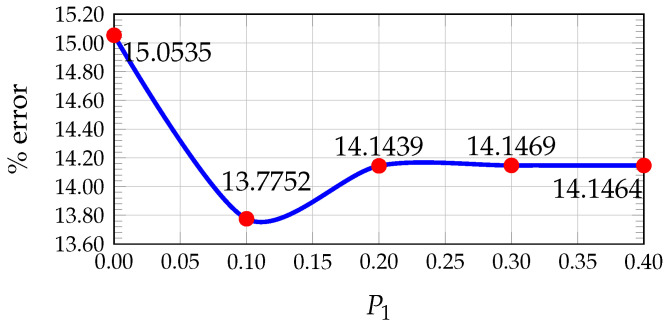
Plot of average percentage error vs. penalty value for 200 images from KITTI.

**Figure 7:**
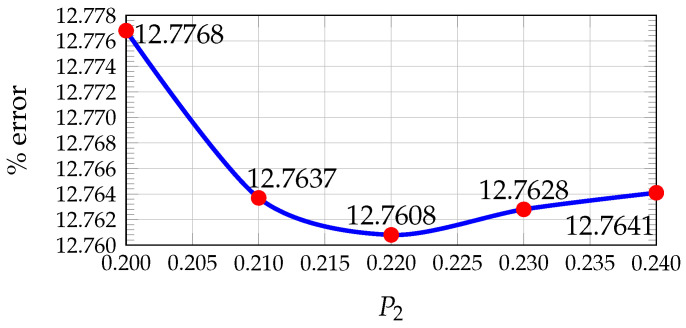
Plot of average percentage error vs. threshold value for occlusions for 200 images from KITTI.

**Figure 8:**
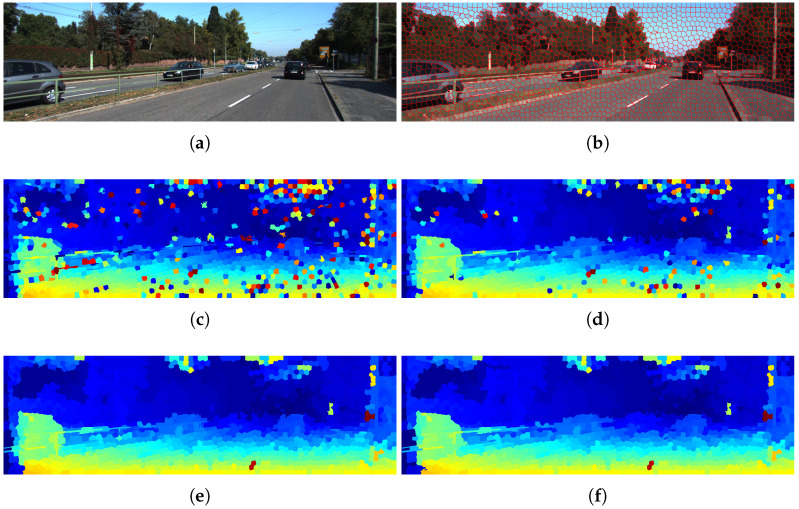
Disparity map calculation process using the proposed methodology on a KITTI image: (**a**) original image; (**b**) SLIC-based segmentation; (**c**) disparity map obtained using the Census Transform descriptor; (**d**) results including an additional color-based criterion $w_{c}$; (**e**) refined disparity map after filtering P1; (**f**) final disparity map after P2. In the disparity maps (**c**–**f**), the color scale represents the magnitude of the disparity, where warmer colors typically indicate higher disparity (closer objects) and cooler colors indicate lower disparity (more distant objects).

The results showed that the optimal values are $w_{c}=0.15$, $P_{1}=0.10$, and $P_{2}=0.22$ (see Figure 5, Figure 6 and Figure 7). Furthermore, Figure 8 illustrates how the disparity calculation improves as each parameter is incorporated. For more details, refer to [24].

The average processing time per frame with sequential programming for the set of 200 images in the KITTI dataset is 23.0780 s with SGM and 10.2151 s with the proposed method using segmented images. On the other hand, SGM achieves a lower error rate of 8.95% on the images tested compared to 12.76% for our proposed method.

### 4.3. Determination of Influences and Free Space Estimation

The computational cost was reduced by applying the Cauchy estimator to the disparity map at the superpixel cluster level. This approach substantially decreases the number of required operations, thereby achieving a significant improvement in processing efficiency. Instead of processing all pixels, the method operates on the centroids of each superpixel, using their spatial coordinates and corresponding disparity values.

The IRLS algorithm is then used to obtain a robust estimation of the ground plane parameters. The weighting scheme includes a scale parameter *c*, which controls the influence of residuals during the iterative optimization. In this work, *c* was set to 2, following common practices in robust regression, to balance sensitivity to inliers and robustness against outliers [27,31]. Additionally, the coplanarity tolerance parameter ϵ was set to 3 according to the three-sigma criterion commonly used in robust estimation, which limits the influence of large residuals while preserving the majority of inlier observations [20].

**Figure 9:**
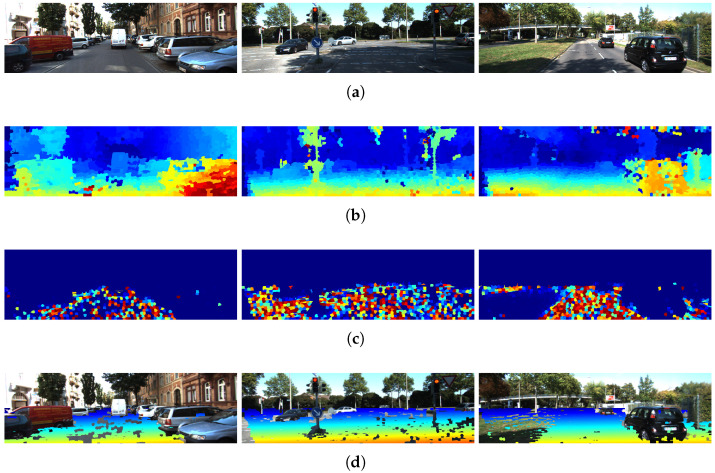
Examples of free space estimation using the plane equation and superpixel influence values for three different KITTI images. (**a**) Original images; (**b**) disparity maps obtained from segmented images; (**c**) influence maps computed using $w\left(r\right)=\frac{1}{1+{\left(\frac{r}{c}\right)}^{2}}$ with c=2; (**d**) initial free space estimation using ground plane information. In (**b**,**d**), the color scale represents the magnitude of the disparity, where warmer colors typically indicate higher disparity (closer objects) and cooler colors indicate lower disparity (more distant objects). In (**c**), warmer colors indicate higher influence, while cooler colors indicate lower influence.

Figure 9 shows examples of the results obtained by applying ground estimation to the disparity maps. From the influence map, each superpixel is weighted according to its influence value to estimate the plane that best fits the ground.

Subsequently, the disparity value at each superpixel is compared with the value predicted by the plane equation. If the difference is within a tolerance of ϵ=±3, the corresponding superpixel is classified as ground, producing a binary mask. Figure 9d shows the resulting ground masks in real scenarios.

### 4.4. Evaluation

To evaluate the proposed methodology, a comparison is performed against two representative approaches that cover fundamentally different paradigms in free space detection. On the one hand, a classical geometric pipeline based on Semi-Global Matching (SGM) combined with the Census Transform (CT) is considered as a widely adopted baseline for stereo-based road understanding. This comparison allows for the impact of the proposed superpixel-based disparity estimation to be assessed within a traditional geometric framework.

On the other hand, the SegFormer-B0 model [8] is included as a representative state-of-the-art deep learning approach for semantic segmentation. This enables a direct comparison between data-driven methods and the proposed geometry-based formulation, highlighting differences in terms of accuracy, generalization capability, and dependency on training data. For this evaluation, four of the most common pixel-level performance measures described in Table 1 are used [32]. In addition, pixels at the edge boundaries of the image are discarded to reduce errors caused by manual ground truth annotation.

**Figure 10:**
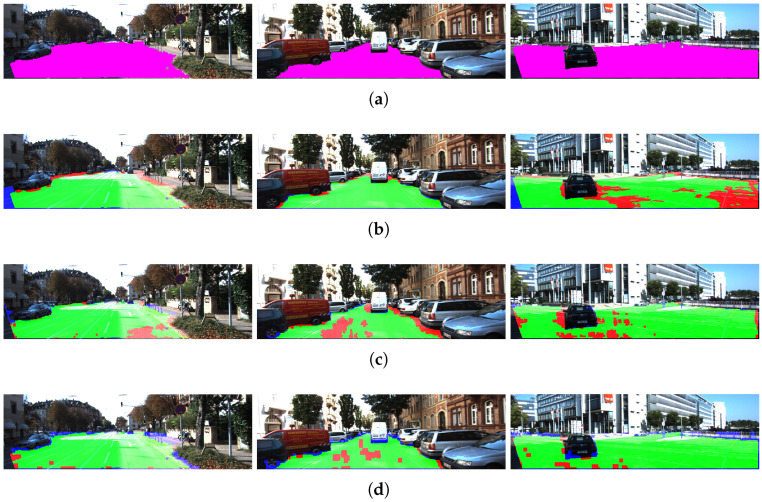
Results on KITTI dataset: (**a**) ground truth of free space, (**b**) estimation using SegFormer-B0, (**c**) estimation using SGM with CT, (**d**) estimation using the proposed model with superpixel clustering. Green indicates True Positives (TP), red indicates False Negatives (FN), blue indicates False Positives (FP), and purple represents the ground truth.

A free space ground truth was generated from the KITTI image dataset through manual segmentation of drivable areas, enabling a comparative evaluation of the three methodologies. Using this ground truth, False Positives (FP), False Negatives (FN), True Positives (TP), and True Negatives (TN) were computed for the set of 200 manually segmented images. The results obtained for the three cases are shown in Table 2. In addition, Figure 10 shows some of the results obtained.

**Figure 11:**
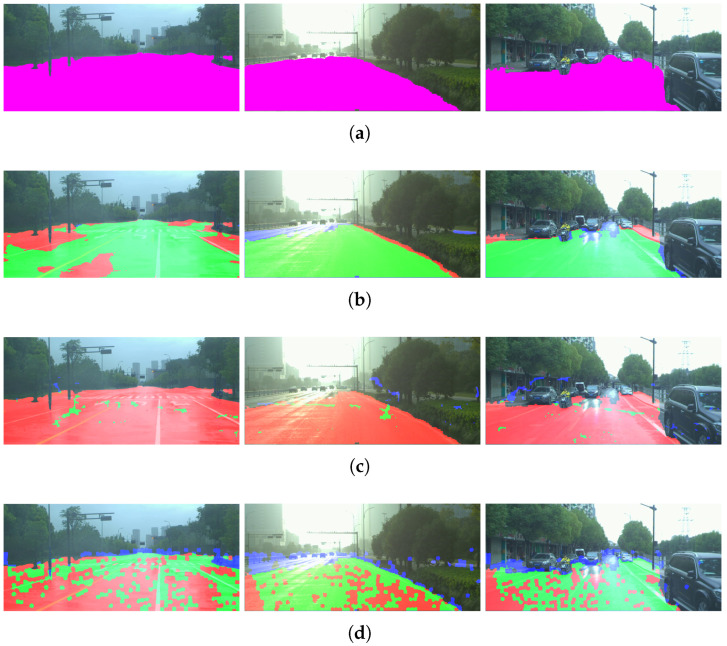
Results on the DrivingStereo dataset: (**a**) ground truth of free space, (**b**) estimation using SegFormer-B0, (**c**) estimation using SGM with CT, and (**d**) estimation using the proposed model with superpixel clustering. The first, second, and third columns correspond to rainy, foggy, and cloudy conditions, respectively. Green indicates True Positives (TP), red indicates False Negatives (FN), blue indicates False Positives (FP), and purple represents the ground truth.

The average results for each weather condition are summarized in Table 3, which reports the performance obtained on the DrivingStereo dataset considering 500 images per weather category (sunny, cloudy, foggy, and rainy), resulting in a total of 2000 evaluated frames. In this case, the ground truth was generated following the same manual segmentation procedure described for the KITTI dataset. In addition, Figure 11 shows some of the results obtained.

## 5. Discussion of Results

In the disparity calculation using images clustered in superpixels, the main result is a 56% reduction in the average processing time per frame. Specifically, for the set of 200 images in the KITTI dataset evaluated with sequential programming, the average processing time decreases from 23.0780 s using SGM to 10.2151 s with the proposed method. On the other hand, SGM achieves an average error rate of 8.95%, compared to 12.76% for the proposed approach, corresponding to a 3.81% increase in the average percentage error. This reduction in processing time is particularly relevant for practical applications, as it demonstrates that the proposed method effectively decreases computational complexity while maintaining competitive detection performance.

It is important to note that the reported runtime corresponds to a non-optimized sequential implementation. From a computational perspective, the proposed methodology presents several characteristics that make it suitable for real-time deployment after optimization.

First, the use of superpixel-based clustering reduces the number of elements involved in disparity estimation, transforming a dense pixel-wise problem into a region-based one. This significantly decreases the number of matching operations and the overall computational burden.

Second, the processing pipeline is inherently parallelizable. The main stages, including superpixel segmentation, disparity estimation for each region, and plane fitting, can be executed independently, enabling efficient implementation on parallel architectures such as GPUs or multi-core processors.

Finally, unlike dense stereo approaches such as SGM, which require global optimization, the proposed method operates locally at the superpixel level, further simplifying the computational process.

These characteristics suggest that, with appropriate optimization and parallel implementation, the proposed method has strong potential to achieve real-time performance in practical applications.

**Figure 12:**
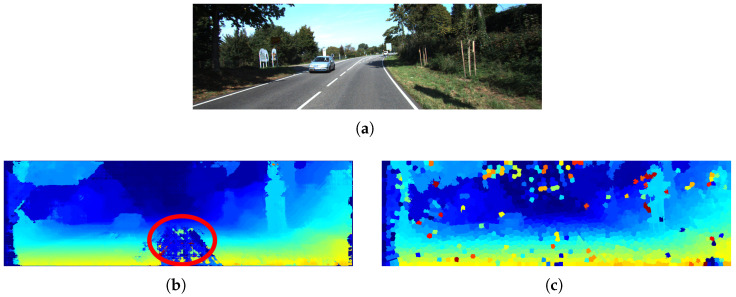
Comparison between disparity maps for a KITTI image: (**a**) original image, (**b**) result using Semi-Global Matching (SGM) with a highlighted error region (indicated by the red circle), and (**c**) result using the proposed method, which shows higher robustness to low-texture surfaces. The color scale in the disparity maps represents the magnitude of the disparity, where warmer colors indicate higher disparity (closer objects) and cooler colors indicate lower disparity (more distant objects).

Additionally, by working with segmented images for disparity estimation, it is possible to incorporate a semi-global criterion that considers not only spatial and texture information but also color space cues, thereby increasing system robustness, as illustrated in Figure 12.

**Figure 13:**
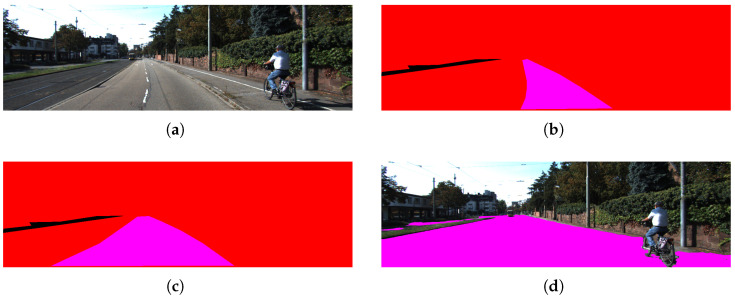
Comparison between different segmentation levels: (**a**) original image from KITTI, (**b**) lane segmentation, (**c**) road segmentation, and (**d**) free space segmentation. In the segmented images (**b**–**d**), the purple color highlights the respective region being segmented (lane, road, or free space).

It is important to emphasize the advantages of free space detection over lane or road detection. Lanes and roads can be considered subsets of free space, as shown in Figure 13, but their detection typically requires visible and well-defined markings. In contrast, free space detection remains applicable even in the absence of lane markings, such as in rural roads, parking lots, unpaved roads, or adverse weather conditions.

The gravity condition assumes that the normal vector to the ground plane is also perpendicular to the XZ plane of the camera coordinate system. This implies that ground detection is reliable only when the terrain does not exhibit significant inclinations.

For this reason, the coplanarity condition is preferred, as it allows the plane normal to adopt different orientations, enabling robust detection on inclined or irregular surfaces. Furthermore, by using plane information from one frame to initialize the next, faster convergence is achieved while maintaining consistent ground tracking.

In addition, operating on segmented images reduces the number of elements required for ground plane estimation. This enables the efficient computation of a plane that separates traversable ground from obstacles and non-drivable regions such as sidewalks or medians.

### Parameter Sensitivity Analysis

The performance of the proposed method depends on a set of key parameters that influence accuracy, robustness, and computational cost. In the following, a systematic analysis of the main parameters is provided.

Superpixel size: The size of the superpixels directly affects both computational cost and accuracy. Smaller superpixels preserve fine structural details and improve boundary adherence, but increase the number of regions to be processed, leading to higher computational cost. In contrast, larger superpixels reduce the number of elements and improve efficiency, at the expense of reduced accuracy near depth discontinuities. Therefore, this parameter defines a trade-off between precision and computational efficiency.

Weighting parameter between structural and color similarity: This parameter controls the balance between the Census Transform (robust to illumination changes) and color information in the CIELAB space. Lower values increase robustness to illumination variations and adverse weather conditions, while higher values improve discrimination in well-lit environments. As a result, this parameter mainly affects robustness under varying environmental conditions.

Robust estimator parameter in plane fitting: The parameter used in the robust estimation process regulates the influence of outliers during plane fitting. Lower values increase robustness by reducing the impact of mismatches and noise, but may discard useful information. Higher values allow more data to contribute to the estimation but reduce robustness. This parameter directly impacts stability in noisy scenarios such as rain or fog.

Coplanarity threshold: This threshold determines whether a pixel is classified as belonging to the ground plane. It has a direct impact on the Precision–Recall trade-off. Larger values increase Recall by including more pixels as free space but also introduce more false positives, reducing Precision. Conversely, smaller values improve Precision but may exclude valid ground regions.

Overall, the results indicate that the proposed method is relatively stable within a reasonable range of parameter values, although it requires further adjustment for extreme environmental conditions such as rain.

Based on the results obtained with KITTI, although the proposed method does not achieve the best results in the Quality (Q), Precision (DR), and Effectiveness (F) metrics, it presents a significant advantage in Recall (DA), reaching a value of 0.9522 compared to 0.8400 for SegFormer-B0 and 0.8901 for CT with SGM. This indicates a strong ability to correctly identify free-space pixels.

However, this behavior is accompanied by a lower Precision (0.7079), indicating the presence of false positives. In safety-critical applications, false positives (i.e., incorrectly identifying obstacle regions as traversable) may pose greater risks than false negatives. Therefore, the observed Precision–Recall trade-off must be carefully considered depending on the intended deployment scenario.

The current configuration favors sensitivity to ground detection, which is beneficial in assisted driving scenarios where overly conservative free-space estimation could unnecessarily restrict maneuverability. Nevertheless, the coplanarity tolerance and decision thresholds can be tuned to increase Precision if required, reducing false positives at the cost of lower Recall. Furthermore, an additional refinement stage or multi-sensor validation could mitigate this limitation in safety-critical implementations.

The increase in false positives directly impacts the Precision (DR) metric, which in turn reduces the Effectiveness (F) score. This suggests that the proposed approach could benefit from an additional refinement stage aimed at reducing false positives without compromising Recall.

In addition to the evaluation on the KITTI dataset, the proposed method was further analyzed using the DrivingStereo dataset under different weather conditions, including sunny, cloudy, foggy, and rainy scenarios.

The results show that learning-based approaches such as SegFormer-B0 consistently achieve higher performance across all weather conditions, due to their ability to learn complex feature representations from large datasets. However, their performance is inherently dependent on the availability of representative training data for each specific condition.

In contrast, the proposed method, while exhibiting lower overall accuracy, maintains a more stable behavior across different weather scenarios without requiring retraining or domain adaptation. This highlights one of the main advantages of the geometric formulation: its independence from training data and its ability to generalize to unseen conditions.

On the other hand, the classical SGM-based approach shows a significant degradation in performance under adverse weather conditions, particularly in foggy and rainy scenarios. This behavior is mainly attributed to the sensitivity of dense stereo matching to low contrast, noise, and visibility degradation.

**Figure 14:**
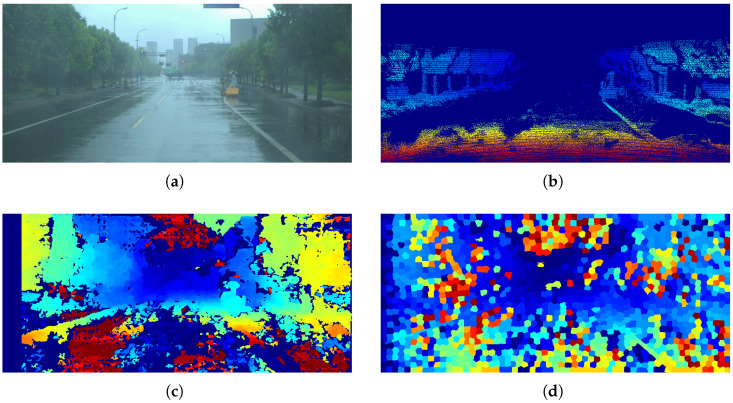
Comparison of the disparity maps obtained: (**a**) original image from DrivingStereo, (**b**) LiDAR disparity map, (**c**) disparity map using SGM, and (**d**) disparity map using the proposed method. In the disparity maps (**b**–**d**), the color scale represents the magnitude of the disparity, where warmer colors indicate higher disparity (closer objects) and cooler colors indicate lower disparity (more distant objects).

It is also important to note that the proposed method exhibits a noticeable decrease in performance under rainy conditions, where the presence of noise and reflections affects disparity estimation and plane fitting. It is worth noting that this problem is not unique to stereographic methods; it also affects LiDAR-based methods, as shown in Figure 14, where the disparity map is affected by rain, according to the DrivingStereo dataset. This limitation suggests that additional preprocessing or refinement stages, such as image restoration or temporal filtering, could further improve robustness in highly degraded environments.

A common limitation of many state-of-the-art methodologies, particularly those based on Convolutional Neural Networks (CNNs), is their strong dependence on large training datasets. In many cases, these datasets are not sufficiently representative to ensure generalization to unseen or atypical scenarios.

Other methodologies rely on predefined seed points or initial ground regions without verifying the actual presence of the ground. This can lead to incorrect free space estimation, particularly if obstacles are present within the assumed ground region.

The proposed methodology addresses these limitations by dynamically estimating the ground plane using the coplanarity condition, allowing for reliable detection even on sloped surfaces. Additionally, the use of segmented images reduces computational cost compared to traditional approaches.

The results demonstrate that the proposed approach maintains stable performance across different environmental conditions without requiring retraining or domain adaptation. However, performance decreases in challenging scenarios such as rain, where noise and reflections affect disparity estimation. Despite this limitation, the method shows robustness compared to classical stereo approaches, although it does not reach the accuracy of learning-based methods.

Nevertheless, the evaluation does not yet cover extreme terrain variations, severe slopes, or nighttime environments. Therefore, further validation on additional datasets is still required to fully assess the generalization capabilities of the proposed method. Future work will focus on extending the evaluation to more diverse scenarios and improving robustness under highly degraded conditions.

It is important to mention that the methodology proposed in this work is performed in several easily separable stages, so when implemented in a parallelizable system, we can resort to a pipeline architecture to optimize the processing time.

Finally, as future work, performance can be improved by reducing false positives and false negatives through an additional refinement stage, opening multiple directions for further research.

## Figures and Tables

**Table 1:** Pixel-wise measures used to evaluate the performance of detection results. These measures are defined using the entries of a contingency table.

Contingency Table			Pixel–Wise Measure	
Result	Ground–truth		Quality	$Q=\frac{TP}{TP+FP+FN}$
	Non–Road	Road	Precision	$DR=\frac{TP}{TP+FP}$
Non–Road	TN	FN	Recall	$DA=\frac{TP}{TP+FN}$
Road	FP	TP	Effectiveness	$F=\frac{2(DR)(DA)}{DR+DA}$

**Table 2:** Average performance of different free space detection algorithms using the full KITTI dataset.

Method	Q	DR	DA	F
SegFormer-B0	0.6914	0.8065	0.8400	0.8073
CT with SGM	0.7898	0.8733	0.8901	0.8778
Our method	0.6825	0.7079	0.9522	0.8047

**Table 3:** Average performance of different free space detection algorithms using the DrivingStereo dataset under various weather conditions.

Weather	Method	Q	DR	DA	F
Sunny	SegFormer-B0	0.6370	0.7101	0.8514	0.8048
	CT with SGM	0.0362	0.3511	0.0667	0.0961
	Our method	0.5493	0.5786	0.6601	0.6955
Cloudy	SegFormer-B0	0.7250	0.8017	0.9006	0.8381
	CT with SGM	0.0338	0.3745	0.0456	0.0833
	Our method	0.3057	0.4942	0.4822	0.5062
Foggy	SegFormer-B0	0.7656	0.8006	0.9520	0.8627
	CT with SGM	0.0302	0.4509	0.0467	0.0796
	Our method	0.3002	0.5219	0.4614	0.5250
Rainy	SegFormer-B0	0.6938	0.9750	0.7021	0.7852
	CT with SGM	0.0320	0.6998	0.0326	0.0613
	Our method	0.2085	0.6954	0.2300	0.3360

## Data Availability

The data availability statement and the links have been added to the public datasets used in this study.

## Abbreviations

The following abbreviations are used in this manuscript:

AI	Artificial Intelligence
CV	Computer Vision
LiDAR	Light Detection and Ranging
NN	Neural Network
CNN	Convolutional Neural Network
3D	three-dimensional
SGM	Semi-Global Matching
CT	Census Transform
MSER	Maximally Stable Extremal Regions
MRF	Markov Random Fields
SLIC	Simple Linear Iterative Clustering
IRLS	Iteratively Reweighted Least Squares
